# Medical-Legal Partnerships Reinvigorate Systems Lawyering Using an Upstream Approach

**DOI:** 10.1017/jme.2023.162

**Published:** 2023

**Authors:** L. Kate Mitchell, Debra Chopp

**Affiliations:** 1: LOYOLA UNIVERSITY CHICAGO SCHOOL OF LAW, CHICAGO, IL, USA; 2: UNIVERSITY OF MICHIGAN LAW SCHOOL, ANN ARBOR, MI, USA

**Keywords:** Medical-Legal Partnership, Upstream, Advocacy, Systemic, Justice

## Abstract

The upstream framework presented in public health and medicine considers health problems from a preventive perspective, seeking to understand and address the root causes of poor health. Medical-legal partnerships (MLPs) have demonstrated the value of this upstream framework in the practice of law and engage in upstream lawyering by utilizing systemic advocacy to address root causes of injustices and health inequities. This article explores upstreaming and its use by MLPs in reframing legal practice.

## Introduction

*Three friends were walking down a path along the side of a river. Suddenly they heard shouting and noticed someone caught up in the current. The friends jumped in the river to save the drowning person. As they saved this person, they could see more drowning people coming downstream towards them. They worked together trying to save as many people as they could, but quickly became overwhelmed and could not keep up. One friend started to gather branches and logs to build a raft which could catch more drowning people, but it was still not enough to save everyone. After some time, another of the friends headed to shore and started walking on the path upstream. In desperation, their two friends called out: “What are you doing? Where are you going? We need help!” The friend yelled back, “I am going upstream to find out how all of these people are getting into the river.”* ^1^

This river parable has been used in public health and medicine to demonstrate the need for an upstream approach to solving pervasive and intractable causes of poor health.^2^ Theories of upstream practice have helped guide public health analysis of social and health-related challenges and problem solving^3^ and have served as a framework for doctors, and now lawyers, to identify and address systemic issues at the root cause of access to care issues, health inequities, and poverty.^4^

Medical-legal partnerships (MLPs) have championed the application of upstream practice in the law by utilizing lawyering strategies that seek to address root causes of poor health through legal care and social and legal harms through systems-based, impact, and policy advocacy. ^5^ These strategies require legal advocates to step away from the common practice of addressing high volumes of individual direct advocacy cases and take the path upstream to identify and address the source of the injustice, harm, or inequity for the benefit of a wider population. For example, if a state Medicaid policy is incompatible with federal law and patients are being denied necessary care to which they are legally entitled, it is more effective to forgo the traditional legal strategy of representing each Medicaid beneficiary individually in separate fair hearing requests and, instead, develop strategies upstream at the source of the problem to correct the policy resulting in care denials for the larger group.^6^ This upstream approach is critical for addressing systemic, pervasive, structural, and generational issues and to stop the otherwise overwhelming flow of individual cases.

This article will explore the origins of the upstream framework and how it has been conceived in public health and medicine. The article will then discuss how MLPs have demonstrated the value of upstreaming in the law and use a case study to showcase how the Pediatric Advocacy Clinic, an MLP at the University of Michigan Law School, has effectively used upstream practices to create systems change.

This article will explore the origins of the upstream framework and how it has been conceived in public health and medicine. The article will then discuss how MLPs have demonstrated the value of upstreaming in the law and use a case study to showcase how the Pediatric Advocacy Clinic, an MLP at the University of Michigan Law School, has effectively used upstream practices to create systems change.

## I. An Overview of Upstreaming

The concept of “upstreaming” has been attributed to various scholars and scientists in public health, social work, medical sociology, medical anthropology, economics, environmental studies, medicine, and law.^7^ In the fields of medicine and public health, this upstream framework has generally been utilized to promote an evaluation of root causes of health challenges or inequities, going beyond the traditional medicalized focus on the symptoms and treatment of the individual.^8^ The upstream framework requires an evaluation of the structural, social, and environmental causes of poor health and thus lends itself to the development of systemic and structural solutions.

Physician John B. McKinlay credited the upstream parable story to medical sociologist Irwin Zola and is known for further developing the theory and introducing it to the field of public health.^9^ McKinlay observed that most resources are focused downstream, warning of the short-term nature and futility of such interventions.^10^ He advocated for more focused attention on “root causes” of illness.^11^

To push the river analogy even further, the task becomes one of furiously swimming against the flow and finally being swept away when exhausted by the effort or through disillusionment with the lack of progress. So long as we continue to fight the battle downstream, and in such an ineffective manner, we are doomed to frustration, repeated failure, and perhaps ultimately a sicker society.^12^

McKinlay argued that even efforts at prevention tend to focus downstream on at-risk behaviors and fail to identify underlying causes of such behaviors.^13^

Johnathon Stoeckle, considered the father of modern primary care, has also been credited with developing a vision for upstreaming in medicine.^14^ A scholar of Dr. Stoeckle’s work notes that as early as 1966, Stoeckle “used the [upstream] metaphor frequently, along with other ways to think about and to intervene in what we came to call the ‘social origins of illness,’ much later leading to the currently favored term ‘social determinants.”^15^ Stoeckle was heavily influenced by community health, nursing, and social work in the evolution of his thoughts on upstreaming.^16^

Since these early discussions of upstreaming in the 1960s, scholars of public health, social work, and medicine have continued to evolve in their focus on root, macro, or distal upstream causes of health inequities, injustices, and poverty.^17^ Upstreaming is often used synonymously, as Stoeckle suggested, with a social determinants of health (SDOH) framework.^18^ The World Health Organization defines SDOH as the “conditions in which people are born, grow, work, live, and age, and the wider set of forces and systems shaping the conditions of daily life.”^19^ These determinants are broad and include societal, community, and structural influencers such as neighborhood safety, environment, education, housing, immigration status, access to care, disability access, racism, financial resources, and so on.^20^ The SDOH are now commonly used as a screening tool in medical practice, public health, and health law and policy as an acknowledgement of the factors that influence health and well-being and as a target of intervention.^21^ The SDOH framework has provided a way to consider the social and structural causes of poor health and health inequities and thus to identifying upstream structural and systemic solutions.^22^

In 2013, Dr. Rishi Manchanda further advanced the concept of integrating SDOH and upstream practices in medicine in *The Upstream Doctors*.^23^ Through a range of case studies, Dr. Manchanda illustrated the benefits of identifying and remedying problems at their source and argued that “the future of health care depends on growing and supporting more ‘upstreamists.’ These are the rare innovators on the front lines of health care who see that health (like sickness) is more than a chemical equation that can be balanced with pills and procedures administered within clinic walls.”^24^ Dr. Manchanda suggested “upstreamists” are innovators in looking to the source of ailments in the places where people “live, work, eat, and play.”^25^ Like Stoeckle, Manchanda links the SDOH to upstreaming, describing them as “significantly more powerful drivers of wellness than is medical care” and explaining that they “are shaped by the power and resources that people have, all of which are influenced by the policy choices we make as a society.”^26^ As Manchanda suggests, this broader understanding of the causes of poor health can provide a path towards changes in practice to address them.

The *American Medical Association’s Organizational Strategic Plan to Embed Racial Justice and Advance Health Equity* for 2021-2022 drew upon Dr. Manchanda’s call for upstream strategies and recognized the framework as essential to understanding the underlying causes of poor health and framing solutions to address health inequities.^27^ In its Strategic Plan, the AMA stated:

Moving *upstream* to address the political, structural and social drivers of health and health inequities, along with working to dismantle the systems of power and oppression that shape these drivers, requires action on the societal, community and individual level.^28^

This call to action from the largest professional organization of physicians reflects a growing acceptance of the wide array of influencers of health and the broader call for strategies to address the systemic and structural causes of poor health.

While upstreaming has become an increasingly recognized framework in public health and medicine, it is also gaining broader traction as a universal model of change. Dan Heath’s 2020 book, *Upstream: The Quest to Solve Problems Before They Happen,* applies the upstream problem-solving strategy to a variety of industries such as education, corporations, and public health challenges.^29^ Heath argued that upstreaming is “a declaration of agency: *I don’t have to be at the mercy of these forces-I can control them. I can shape my world.* And in that declaration are the seeds of both heroism and hubris.”^30^ As Heath suggests, the upstream framework is ripe for use by a wide variety of professions because it lends itself to creative source-based solutions.

## II. Medical-legal Partnerships Bring Upstreaming to the Practice of Law

While practitioners in medicine and public health have embraced upstream frameworks in conceptualizing solutions targeted at root causes of poor health, injustice, or inequities, MLPs have demonstrated the effective application of upstreaming in the practice of law. MLPs both operate as an upstream method of healthcare delivery by addressing the legal and social causes of poor health at the individual patient level, and as a model for upstreaming as a practice in law by utilizing strategies to address the sources, policies, practices, and structures, that are at the root cause of the health harming legal issues they address.^31^

MLPs engage in upstreaming by forming interdisciplinary partnerships with health care providers to identify root causes of poor health and working collaboratively to address them. Through these partnerships, MLPs use legal advocacy to address SDOH by representing individual clients in cases impacting safety, public assistance, safe housing, access to health care, accommodations at school, etc.^32^ For example, if a patient repeatedly presents to the emergency department with symptoms of asthma, the downstream method of patient care may include prescribing breathing treatments for the patient.^33^ An upstream approach is to ask the patient about the conditions in their home or environment that could be causing or exacerbating the breathing trouble, and then prescribing a lawyer to address that problem, possibly by sending a demand letter or by suing the patient’s landlord to remedy unsafe conditions in the home.^34^

MLPs also engage in lawyering further upstream by addressing structural determinants of health and correcting systemic issues that have broader effects on recurring individual challenges affecting health and wellbeing.^35^ To revisit the example relating to housing conditions, this might mean using legal advocacy to file a broader impact case on behalf of similarly situated tenants, or by engaging in policy advocacy to change laws and policies governing housing conditions.^36^ Through these more systemic upstream strategies, a lawyer can address the health-harming condition further upstream at the point of the structure, policy, or practice.

Among MLP scholars, this systemic upstream approach has been called patients-to-policy,^37^ public health,^38^ population health,^39^ health justice,^40^ and health equity^41^ approaches. Scholars have called for an upstream approach to address a variety of structural problems such as the opioid epidemic,^42^ childhood trauma,^43^ and the enforcement of the guarantees of children’s Medicaid.^44^ These upstream frameworks are critical to prevent the constant downstream flow of crisis after crisis. By engaging in upstream work, MLPs strengthen the practice of law by modeling a new framework for promoting structural change. This systems-based approach is critical for addressing poverty, oppression, racism, and other structural and systemic drivers of poor health and injustice.^45^

## III. Upstreaming in Practice: A Case Study

An example of the power of MLPs to work upstream comes from the Pediatric Advocacy Clinic (PAC), an MLP between the University of Michigan Healthcare System (“Michigan Medicine”) and the University of Michigan Law School.^46^ Law students enrolled in the clinic represent low-income families on legal issues connected to child health and wellbeing under the supervision of clinical faculty at the Law School. The PAC has embraced upstream practice and successfully addressed systemic problems at their source.

When students in PAC met Dawn, she was the second client referred for assistance getting school support for a do-not-resuscitate (DNR) order for a terminally ill child. Dawn’s son, Willy, had a degenerative neurological illness and received treatment from the palliative care team at the University of Michigan Children’s Hospital. Willy attended school every day, but Willy’s school district had a policy that it would not implement DNR orders without a court order.^47^ Practically speaking, this meant that should Willy suffer cardiac arrest or respiratory failure at school, school personnel would call 911 and attempt resuscitation while waiting for the paramedics. Willy had a valid DNR order - signed by his parents and physicians instructing caregivers and medical personnel to refrain from attempting resuscitation - because they agreed that resuscitation efforts would only cause Willy pain in his final moments of life.

The PAC had seen this issue before when it represented the parents of a terminally ill adult child over whom they had guardianship in a similar case. In that case, the school district had also required a court order to comply with the student’s DNR. That school district expressed that it did not actually oppose DNR orders — in fact, it was the school’s lawyer who referred the family to the clinic — it simply required a court to approve the DNR order’s application in the school setting due to district policy and fear of liability. In this prior case, the PAC asked the circuit court to require the school district to honor the DNR order and rule that the school district’s policy violated a parent’s constitutional right to raise and direct the medical care of their child. The court opted not to address the broader constitutional challenge to the district policy and only addressed the DNR order for that one student, leaving the problematic policy in place for future families to deal with.^48^

In Dawn’s case, the PAC filed a complaint for declaratory judgment asking the court to require the school district to follow the instructions in Willy’s DNR order and asking the court to find the school district’s policy to be an unconstitutional infringement on Willy’s parents’ rights.^49^ Unfortunately, Willy died in the course of litigation — he died peacefully, at home with his family — and the clinic withdrew the case. The case didn’t end with Willy’s death, however. Instead, it inspired upstream advocacy.

When Willy died, PAC students, trained in upstream problem-solving, decided to reassess the clinic’s legal strategy and explore the issue more broadly to understand the scope of the problem. Dawn was supportive and determined that other parents should not go through months of litigation to enforce their right to spare their child pain in their final moments of life.

As part of their upstream evaluation, law students sent Freedom of Information Act (FOIA) requests to every intermediate school district in the state of Michigan. The goal was to learn how student DNR orders were handled throughout the state. When the responses came in, it became clear that different policies existed throughout the state. Many school districts gave the superintendent authority to decide whether to follow the instructions of a DNR order, some set out requirements for the superintendent to make that decision, others required a committee or prohibited the honoring of DNR orders outright.^50^ The discrepancies among districts made it challenging for vulnerable families and providers to know what to expect with regard to sensitive end-of-life decisions for terminally ill students.

By collaborating with healthcare professionals, MLPs also educate the healthcare profession about the power of interdisciplinary efforts to address systemic drivers of poor health. MLPs are therefore uniquely positioned to partner with health care providers to push upstream to achieve structural change.

Michigan’s Do Not Resuscitate Procedures Act (DNR Act) did not make things any clearer. The DNR Act specified the requirements of executing a valid DNR order and specified the locations outside of the hospital in which the orders must be implemented.^51^ However, the DNR Act was silent as to its application in schools.

Recognizing a problematic gap in the DNR Act, the PAC teamed up with palliative care doctors and nurses, as well as the superintendent of the school district Dawn’s son had attended, to launch a statewide advocacy initiative seeking a law clarifying the obligation of school districts to comply with DNR orders for students. PAC students reached out to stakeholders across the state — from disability rights groups to right-to-life organizations and school administrators. They drafted proposed language to amend the DNR Act and the Michigan Revised School Code. Together with a palliative care doctor and nurse as well as their client Dawn, they arranged for and attended meetings with members of the state legislature who were interested in cosponsoring these bills. After years of advocacy by the PAC and palliative care team as well as legislative testimony from Dawn and the director of the PAC, the governor signed a bill requiring that school districts implement properly executed DNRs for students.^52^

This successful advocacy effort, which united a broad coalition of professionals dedicated to improving the health and well-being of critically ill students and their families, underscores the power of MLPs engaged in upstream advocacy. The PAC and its partners corrected a recurring burden for the parents of terminally ill children by identifying and addressing the source of inconsistencies in school district policies, the state DNR Act’s silence on its application in schools.

This upstream approach is applicable to innumerable other advocacy areas as well. For example, the PAC represented several children over multiple years who were having trouble getting continuous glucose monitors (CGM) that help track blood glucose for their Type I diabetes covered by Medicaid managed care companies. The PAC experienced success in obtaining approval for CGM coverage by filing fair hearing requests on behalf of individual families and arguing that CGMs are medically necessary to manage the disease and should be covered for children as a matter of federal Medicaid law.^53^ To address the problem upstream, the PAC collaborated with the pediatric endocrinology team at Michigan Medicine as well as the American Diabetes Association, to pressure the state Department of Health and Human Services to change its Medicaid policies around CGM coverage for minors. The pediatric endocrinologists compiled data regarding the necessity of CGM use as part of effective diabetes management, the PAC wrote a legal memo about the requirements in federal law to cover medically necessary treatment for children who have Medicaid, and the American Diabetes Association leveraged its connections with the State Department of Health and Human Services to turn these changes into a new policy which recognized CGMs for children as a covered service under state Medicaid policy.^54^ Rather than continuing to fight for CGM coverage patient-by-patient, this multidisciplinary, upstream effort ensured improved healthcare for children with diabetes throughout the state of Michigan.

These examples from the PAC demonstrate the value of upstream lawyering. Without utilizing upstream strategies, PAC students and faculty would be frustrated, caught up in the river of filing preliminary injunctions for pediatric DNR cases and one fair hearing request after another to obtain approval for CGMs for children with diabetes. To prevent these challenges from negatively impacting children and parents in the future, it is critical for advocates to step out of the swirling current and walk upstream to explore the root source of the issues impacting the lives of patients and clients.

## IV. Conclusion

*There comes a point where we need to stop just pulling people out of the river. We need to go upstream and find out why they are falling in.*

- Desmond Tutu, Nobel Peace Prize laureate, South African human rights and justice leader.^55^

In some sense, engaging in legal work with the goal of affecting a large number — or a class — of people is not a new concept. Poverty lawyers have employed impact advocacy and policy work as tools to address systemic harms since the origins of poverty law.^56^ The use of the upstream approach by MLPs builds on the legacy of impact legal work and pushes the legal profession to consider the root systemic causes of issues that affect the health and well-being of vulnerable clients every day. By collaborating with healthcare professionals, MLPs also educate the healthcare profession about the power of interdisciplinary efforts to address systemic drivers of poor health. MLPs are therefore uniquely positioned to partner with health care providers to push upstream to achieve structural change.

## Note

The authors have no conflicts to disclose.

## References

[1] See D. Heath , Upstream: The Quest to Solve Problems Before They Happen (New York: Avid Readers Press, 2020): 1 (adapted public health parable, attributed to medical sociologist Irving Zola); R. Manchanda, *The Upstream Doctors* (New York: TED, 2013): Prologue (This parable is a retelling by authors.)

[2] *Id.*

[3] *Id.*; W. Zelman and G. D. Stevens , “Public Health: Swimming Upstream in Search of Influence,” Health Affairs Forefront, July 9, 2020; N. Krieger, “Proximal, Distal, and the Politics of Causation: What’s Level Got to Do With It?” *American Journal of Public Health* 98, no. 2 (2008): 221-230. 10.2105/AJPH.2007.111278 PMC2376874 18172144

[4] J. Teitelbaum , “Obligation and Opportunity: Medical-Legal Partnership in the Age of Health Reform,” Journal of Legal Medicine 35 (2014): 7–28. 24669806 10.1080/01947648.2014.884429

[5] Y. Cannon , “Closing the Health Justice Gap: Access to Justice in Furtherance of Health Equity,” Columbia Human Rights Law Review 53, no. 2 (2021): 517–581; E. Tobin-Tyler and J. B. Teitelbaum, “Medical-Legal Partnership: A Powerful Tool for Public Health and Health Justice,” *Public Health Reports* 134, no. 2 (2019): 201-205; E. Tobin-Tyler, “Medical-Legal Partnership in Primary Care: Moving Upstream in the Clinic,” *American Journal of Lifestyle Medicine* 13, no. 3 (2017): 282-291. 10.1177/1559827617698417 PMC6506975 31105492

[6] L. Kate Mitchell , “The Promise and Failures of Children’s Medicaid and the Role of Medical-Legal Partnerships as Monitors and Advocates,” Health Matrix: Journal of Law-Medicine 30, no. 1 (2020): 175–231.

[7] E. Benfer et al., “Setting the Health Justice Agenda: Addressing Health Inequity and Injustice in the Post-pandemic Clinic,” Clinical Law Review 28 (2021): 45–84; Cannon, * supra * note 5.

[8] J. B. McKinlay , “A Case for Refocusing Upstream: The Political Economy of Illness,” Interdisciplinary Association for Population Health Science, November 18, 2019, at 1 (originally published in 1975 in “Applying Behavioral Science to Cardiovascular Risk,” *American Heart Association: Applying Behavioral Science to Cardiovascular Risk*, reprinted with permission); P. G. Butterfield, “Upstream Reflections on Environmental Health: An Abbreviated History and Framework for Action,” *Advances in Nursing Science* 25, no. 1 (2002): 32-49; S. Reed, E. J. Evans, and N. Hooyman, “Social Work: Leading the Move Upstream to Improve the Nation’s Health,” *Health & Social Work* 45, no. 2 (2020): 77-79. 10.1093/hsw/hlaa006 32355955

[9] See McKinlay, *supra* note 8.

[10] *Id.* at 6.

[11] *Id.*

[12] *Id.*

[13] *Id.*

[14] H. Waitzkin , “John D. Stoeckle and the Upstream Vision of Social Determinants in Public Health,” American Journal of Public Health 106, no. 2 (2016): 234–236. 26691111 10.2105/AJPH.2015.302936 PMC4815816

[15] See Waitzkin, *supra* note 14, at 234 (citing H. Waitzkin , “The Social Origins of Illness: A Neglected History,” International Journal of Health Services 11, no. 1 (1981): 77–103. 7016768 10.2190/5CDV-P4FE-Y6HN-JACD

[16] See Waitzkin, *supra* note 14, at 235.

[17] See Kreiger, *supra* note 3; “The Root Causes of Health Inequity,” in A. Baciu , Y. Negussie , and A. Geller , eds., Communities in Action: Pathways to Health Equity (Washington D.C.: National Academies Press, 2017): 38–41. 28418632

[18] P. Braverman and L. Gottlieb , “The Social Determinants of Health: It’s Time to Consider the Causes of the Causes,” Public Health Reports 129 (2014): 19–31. 10.1177/00333549141291S206 PMC3863696 24385661

[19] *Social Determinants of Health*, World Health Organization, *available at* <https://www.who.int/health-topics/social-determinants-of-health#tab=tab_1> (last visited November 20, 2023).

[20] *Id.*; B. Krishnamurthy et al., “What We Know and Need to Know about Medical-Legal Partnership,” South Carolina Law Review 67, no. 2 (2016): 377–388).

[21] *Id.*; A. Andermann , “Screening for Social Determinants of Health in Clinical Care: Moving from the Margins to the Mainstream,” Public Health Review 39, no. 19 (2018): 1–17. 10.1186/s40985-018-0094-7 PMC6014006 29977645

[22] Braverman and Gottlieb, *supra* note 18.

[23] Manchanda, *supra* note 1.

[24] *Id.* at 5.

[25] *Id.*

[26] *Id.* at 19.

[27] *AMA’s Organizational Strategic Plan to Embed Racial Justice and Advance Health Equity*, American Medical Association, *available at* <https://www.ama-assn.org/about/leadership/ama-s-strategic-plan-embed-racial-justice-and-advance-health-equity> (last visited November 20, 2023) [hereinafter cited as AMA Strategic Plan].

[28] *Id.* at 18.

[29] See Heath, *supra* note 1.

[30] *Id.* at 15.

[31] E. Tobin-Tyler and E. Paul, *Medical-legal partnership builds a culture of upstream advocacy*, Gold Foundation, *available at* <https://www.gold-foundation.org/medical-legal-partnership-builds-a-culture-of-upstream-advocacy> (last visited November 20, 2023); Manchanda, *supra* note 1.

[32] MLPs “embed lawyers on the health care team to help health centers treat patients’ immediate social needs and also deploy upstream strategies to address the social determinants of health.” Kate Marple et al., *Toolkit: A planning, implementation, and practice guide for building and sustaining a health center-based MLP*, National Center for Medical Legal Partnership, *available at* https://medical-legalpartnership.org/mlp-resources/health-center-toolkit (Oct. 6, 2020).

[33] M. M. O’Sullivan et al., “Environmental Improvements Brought by the Legal Interventions in the Homes of Poorly Controlled Inner-city Adult Asthmatic Patients: A Proof-of-Concept Study *,”* Journal of Asthma 49, no. (2012): 911–917; B. Zuckerman, “Medicine and Law: New Opportunities to Close the Disparity Gap,” *Pediatrics* 130, no. 5 (2012): 943-944. 10.3109/02770903.2012.724131 23020301

[34] O’Sullivan et al., *supra* note 33.

[35] Krishnamurthy et al., *supra* note 20.

[36] A. F. Beck , et al., “Identifying and Treating a Substandard Housing Cluster Using a Medical-Legal Partnership,” Pediatrics 130, no. 5 (2012): 831–838; E. A. Benfer, “Contaminated Childhood: How the United States Failed to Prevent the Chronic Lead Poisoning of Low-Income Children and Communities of Color,” *Harvard Environmental Law Review* 41 (2017): 493-561. 23090340 10.1542/peds.2012-0769

[37] See Tobin-Tyler and Paul, *supra* note 31.

[38] E. M. Lawton and M. Sandel , “Medical-Legal Partnerships: Collaborating to Transform Healthcare for Vulnerable Patients,” Journal of Legal Medicine 35, no. 1 (2014): 1–6. 24669805 10.1080/01947648.2014.884426

[39] E. Benfer et al., *supra* note 7.

[40] Cannon, *supra* note 5; E. Tobin-Tyler , “Aligning Public Health, Health Care, Law and Policy: Medical-Legal Partnership as a Multilevel Response to the Social Determinants of Health,” Journal of Health and Biomedical Law 8 (2012): 211–214.

[41] E. Benfer et al., “Health Justice Strategies to Combat the Pandemic: Eliminating Discrimination, Poverty, and Health Disparities During and After COVID-19,” Yale Journal of Health Law, Policy, and Ethics 19, no. 3 (2020): 122–171.

[42] Y. Cannon , “The Kids Are Not Alright: Leveraging Existing Health Law to Attack the Opioid Crisis Upstream,” Florida Law Review 71, no. 3 (2019): 765–830.

[43] Y. Cannon , “Disrupting the Path from Childhood Trauma to Juvenile Justice: An Upstream Health and Justice Approach,” Fordham Urban Law Journal 43, no. 3 (2016): 425–493.

[44] Mitchell, *supra* note 6.

[45] J. Teitelbaum and E. Lawton , “The Roots and Branches of the Medical-Legal Partnership Approach to Health: From Collegiality to Civil Rights to Health Equity,” Yale Journal of Health Law, Policy, and Ethics 17, no. 2 (2017): 344–377; S. Foster , Y. Cannon , and G. Bloche , “Health Justice is Racial Justice: A Legal Action Agenda for Health Disparities,” *Health Affairs*, *available at* <https://www.healthaffairs.org/content/forefront/health-justice-racial-justice-legal-action-agenda-health-disparities> (last visited November 20, 2023).

[46] *Pediatric Advocacy Clinic*, University of Michigan School of Law, *available at* <https://michigan.law.umich.edu/academics/experiential-learning/clinics/pediatric-advocacy-clinic> (last visited November 20, 2023).

[47] Policy on file with the Pediatric Advocacy Clinic; Dawn Renee Krause, “Do-Not-Resuscitate Orders, Michigan Schools, and One Boy’s Journey with His Family,” Legislative Issues no. 58, *available at* <https://www.nhpco.org/wp-content/uploads/Issue-58-Final-1.pdf>, at 5 (last visited November 20, 2023).

[48] *Washtenaw Intermediate School District v. Kathy and Paul Dobrowolski*, 14-626 OZ (on file with Pediatric Advocacy Clinic).

[49] Krause, *supra* note 47; *Dawn Krause and Todd Pickett v. Washtenaw Intermediate School District*, 15-233 CZ (on file with Pediatric Advocacy Clinic).

[50] FOIA request and records on file with Pediatric Advocacy Clinic.

[51] MCL § 333.1051 et. seq. (2020).

[52] MLC § 33.1051 et. seq. (2023); 2020 Mich. Pub. Acts 358, *available at* <https://www.legislature.mi.gov/documents/2019-2020/publicact/pdf/2020-PA-0363.pdf> (last visited November 20, 2023).

[53] 42 U.S.C. §§ 1396a(a)(10), 1396a(a)(43), 1396d(a)(4)(B), 1396d(r)(5) (2019); “Early and Periodic Screening, Diagnostic, and Treatment,” Medicaid, *available at* <https://www.medicaid.gov/medicaid/benefits/early-and-periodic-screening-diagnostic-and-treatment/index.html> (last visited November 20, 2023).

[54] *Michigan Medicaid Continuous Glucose Monitor Policy Proposal*, University of Michigan Health C.S. Mott Children’s Hospital Pediatric Diabetes, *available at* <https://www.umpedsdiabetes.com/policy-advocacy> (last visited November 20, 2023); *Michigan Medicaid Policy Bulletin 23-31: Revisions to Continuous Glucose Monitoring Systems (CGMS) Policy*, Michigan Department of Health and Human Services, *available at* <https://www.michigan.gov/mdhhs/-/media/Project/Websites/mdhhs/Assistance-Programs/Medicaid-BPHASA/2023-Bulletins/Final-Bulletin-MMP-23-31-CGM.pdf?rev=0ec25e040bfd4ca992b7be208cbf2ed4&hash=E6341D3AB94363AC332DBE2EFB11E44D> (issued May 1, 2023; effective June 1, 2023).

[55] See AMA Strategic Plan, *supra* note 27.

[56] D. J. Cantrell , “A Short History of Poverty Lawyers in the United States,” Loyola Journal of Public Interest Law 5 (2003): 11–36.

